# Trial by YouTube: effects of expert psychiatric witness testimony on viewers' opinions of Amber Heard and Johnny Depp

**DOI:** 10.1192/bjb.2024.31

**Published:** 2025-02

**Authors:** Oliver Mason, Beth Horton, Caitlin Starrett

**Affiliations:** University of Surrey, Guildford, UK

**Keywords:** Psychiatry and law, stigma and discrimination, personality disorders, substance use disorders, experimental design

## Abstract

**Aims and method:**

We aimed to assess whether viewing expert witness evidence regarding the mental health of Johnny Depp and Amber Heard in the 2022 court case in the USA would affect viewers’ attitudes towards the mental health of the two protagonists and towards mental illness in general. After viewing excerpts of the cross-examination evidence, 38 trial-naive undergraduate students completed the Prejudice towards People with a Mental Illness (PPMI) scale.

**Results:**

Following viewing, participants held more stigmatising views of the protagonists than they held about mental disorders in general.

**Clinical implications:**

It is plausible that mass media trial coverage further stigmatises mental illness.

Amber Heard and Johnny Depp divorced acrimoniously in 2016 amid allegations of sexual, physical and emotional abuse made by Heard. Two court cases later took place in the UK and USA respectively. The first was heard in the High Court in London, where Depp lost his libel case against the *Sun* newspaper. However, in the subsequent case Depp brought in the Virginia courts in respect of Heard's 2018 ‘op-ed’ piece in *The Washington Post*,^1^ Heard's evidence was roundly rejected by a jury. In contrast to the UK trial, the trial in Virginia was livestreamed globally on YouTube and led to extensive coverage across mainstream and social media. YouTube's Law and Crime channel has seen more than a billion views, making it the most watched trial in history. As part of this trial both teams called expert witnesses to testify in relation to the mental health status of both protagonists – notably, the forensic psychologist Dr Shannon Curry and psychiatrist Dr David Spiegel. From examination of multiple sources of evidence and assessments of Heard, Curry claimed support for diagnoses of borderline personality disorder and histrionic personality disorder. Spiegel testified with a ‘degree of medical certainty that Depp had narcissistic personality traits’, had problems consistent with a substance use disorder and ‘is a perpetrator of intimate-partner violence’.^2^ The socially proscribed behaviour of both protagonists to support these labels were described in great detail in court during expert witness cross-examination, with the aim of discrediting them and their testimony to the jury. The only study to date about the effects of the trial found evidence that global Twitter content referenced the three relevant personality disorder terms in more stigmatising terms for nearly 2 months following the trial,^3^ suggesting an effect of the trial on public opinion more generally.

We were interested in the effects of viewing YouTube court recordings of the presentations by mental health professionals concerning Heard and Depp on attitudes towards their mental health. A systematic review of media reporting of severe mental illness, perhaps unsurprisingly, concluded ‘that positive news reports and social media posts are likely to lead to reductions in stigmatizing attitudes and negative reports and social media posts are likely to increase stigmatizing attitudes’.^4^ We hypothesised that trial coverage of the manner in which evidence was presented by expert witnesses regarding the mental state of Heard and Depp would produce highly negative attitudes in respect of their mental health and thereby further increase stigmatising attitudes regarding mental illness.

## Method

A within-subjects repeated measures design saw participants complete measures concerning their attitudes to mental illness both before and after viewing video coverage of expert witness testimony concerning either Heard or Depp. We used convenience sampling to recruit undergraduate students who had not previously viewed any trial material (*n* = 38; 75% female; age range 20–23 years, mean 21.5 years, s.d. = 0.89). After reading the participant information sheet and giving written informed consent, participants completed the 28 items of the Prejudice towards People with a Mental Illness (PPMI)^5^ scale. The PPMI scale has four subscales measuring dimensions underlying prejudice: Fear/avoidance (fear of people with mental illness and the desire for social distance from them; α = 0.89), Malevolence (lack of sympathy for people with mental illness and belief in their inferiority; α = 0.73), Authoritarianism (belief in the need to coercively control people with mental illness; α = 0.72) and Unpredictability (belief that the behaviour of people with mental illness is unpredictable; α = 0.72).^5^ Participants then viewed approximately 15 min of material taken directly from the livestream of the trial on the YouTube Law and Crime channel (www.youtube.com/@LawAndCrime). Participants were randomly given coverage of either Curry's or Spiegel's testimony and supporting materials; 20 viewed coverage of Heard/Curry and 17 viewed coverage of Depp/Spiegel. We aimed that both sets of materials gave a clear and comprehensive account of coverage of the manner in which evidence was presented by expert witnesses regarding the mental health of both protagonists using material made available to them to the same degree of detail and using the same length of trial video excerpts. Following viewing, participants completed the PPMI reworded so that references to mental illness were replaced by the name of Amber Heard or Johnny Depp (e.g. ‘I would be less likely to become romantically involved with people like Amber Heard’; details of all materials are available from the corresponding author on request).

The authors assert that all procedures contributing to this work comply with the ethical standards of the relevant national and institutional committees on human experimentation and with the Helsinki Declaration of 1975, as revised in 2008. All procedures involving human participants were approved by University of Surrey Ethics Committee (956732-956714-101581455).

## Results

PPMI results for both groups before and after viewing the trial excerpts are given in Table 1, together with significant findings when tested by repeated-measures analysis of variance (ANOVA). There was a very marked increase in stigmatising attitudes towards mental illness after viewing, with 33% of the variance in attitude change explained by the within-subjects factor of exposure to trial materials. Repeated-measures ANOVAs produced the same significant pattern of results for the subscales of Fear, Malevolence and Unpredictability, but not for Authoritarianism. Ratings on the PPMI of Heard were no different from those of Depp on *post hoc* testing.

**Table 1:** Ratings and analyses of variance (within-subjects effects) on the Prejudice towards People with Mental Illness (PPMI) scale before and after viewing excerpts of the Depp *v* Heard trial

PPMI item	Amber Heard rating, mean (s.d.)		Johnny Depp rating, mean (s.d.)		*F*(1,36)	η^2^
	Before	After	Before	After		
Total Prejudice	57.0 (8.7)	71.9 (11.6)	59.1 (9.6)	72.6 (11.5)	90.3***	0.33
Fear	15.3 (4.3)	25.0 (5.5)	16.6 (4.6)	23.8 (6.6)	128.0***	0.78
Malevolence	10.8 (3.3)	14.8 (3.5)	11.6 (3.6)	14.8 (4.4)	25.4***	0.20
Unpredictability	18.4 (3.4)	20.4 (3.8)	19.9 (3.4)	21.7 (4.0)	13.1***	0.06
Authoritarianism	12.7 (3.3)	11.7 (2.9)	11.0 (3.8)	12.3 (4.1)	0.13	0.0

*** *P* < 0.001.

## Discussion

The coverage of the mental health and possible mental disorders (in the opinion of expert witnesses) of both Heard and Depp appeared to lead to our admittedly small sample forming very prejudicial views – more negative than the participants’ previously held views about mental illness in general. Although it is not possible from our study to say whether the negative views formed of trial protagonists affect people's perceptions of mental illness in general, the fact that diagnostic labels were used and elaborated on by expert witnesses as part of their role in discrediting testimony, tarnishing reputation and thereby undermining the credibility of both parties is highly concerning. Case-naive participants viewing the expert testimony for the first time formed prejudicial views about both parties – interestingly, these were highly similar for both individuals. Although the experts may have used mental illness labels to stereotype Heard and Depp by association, the effect on the viewing public may well be that more prejudiced views not only of these individuals but also concerning mental disorder more generally result. This is consistent with the wider literature on media reporting of mental illness^4^ and with the linguistic analysis of Twitter posts about mental illness, and personality disorder in particular, following the trial.^3^ The convenience sample of students limits conclusions as to the wider generalisability of our findings. Also, as a result of our study design it is not possible to determine whether the higher scores post-viewing are indicative of increased stigmatising attitudes towards mental illness as a whole, rather than simply towards Heard and Depp.

## Data Availability

The data that support the findings of this study are available from the corresponding author, O.M., on reasonable request.

## About the authors

**Oliver Mason** (Reader in Psychology), **Beth Horton** (undergraduate student) and **Caitlin Starrett** (undergraduate student) were affiliated with the School of Psychology at the University of Surrey, Guildford, UK, during this study.

## References

[1] Heard A. ‘I spoke up against sexual violence – and faced our culture's wrath. That has to change’. The Washington Post, 2018: 18 Dec (https://www.washingtonpost.com/opinions/ive-seen-how-institutions-protect-men-accused-of-abuse-heres-what-we-can-do/2018/12/18/71fd876a-02ed-11e9-b5df-5d3874f1ac36_story.html).

[2] *Depp v. Heard*, CL-2019-2911 (VA Cir. Ct 27 Mar 2020).

[3] Fang A, Zhu H. Measuring the stigmatizing effects of a highly publicized event on online mental health discourse. In CHI ’23: *Proceedings of the 2023 CHI Conference on Human Factors in Computing Systems* (eds S Barbosa, C Lampe, C Appert, DA Shamma, S Drucker, J Williamson, et al.): Article 482. Association for Computing Machinery, 2023.

[4] Ross AM, Morgan AJ, Jorm AF, Reavley NJ. A systematic review of the impact of media reports of severe mental illness on stigma and discrimination, and interventions that aim to mitigate any adverse impact. Soc Psychiatry Psychiatr Epidemiol 2019; 54: 11–31. 30349962 10.1007/s00127-018-1608-9

[5] Kenny A, Bizumic B, Griffiths KM. The Prejudice towards People with Mental Illness (PPMI) scale: structure and validity. BMC Psychiatry 2018; 18(1): 293. 30223823 10.1186/s12888-018-1871-z PMC6142319

